# A Prospective Observational Study Comparing Retrorectus and Anterectus Mesh Placement in Incisional Hernia Repair

**DOI:** 10.7759/cureus.84893

**Published:** 2025-05-27

**Authors:** Havil Stephen Alexander Bakka, Prathibha Kamepalli, Kayaththery Varathan, Adele Zacken, Tharaga Kirupakaran, Daniel I Koshy, Sanjeevi Bharadwaj, Suryanarayana Bandlamudi

**Affiliations:** 1 Neurosurgery, Royal Sussex County Hospital, Brighton, GBR; 2 General Surgery, NRI Medical College & General Hospital, Vijayawada, IND; 3 Orthopaedics and Trauma, Barts Health NHS Trust, London, GBR; 4 General Surgery, Barking, Havering and Redbridge University Hospitals NHS Trust, London, GBR; 5 Acute Medicine, Kingston Hospital NHS Foundation Trust, Kingston, GBR; 6 Trauma and Orthopaedics, Royal London Hospital, London, GBR; 7 Orthopaedics and Traumatology, National Health Service (NHS), Birmingham, GBR

**Keywords:** abdominal wall surgery, anterectus repair, hernia recurrence, hernioplasty, incisional hernia, mesh placement, postoperative complications, retrorectus mesh repair, surgical outcomes, surgical outcomes research

## Abstract

Background

Incisional hernias are a frequent complication following abdominal surgeries, significantly contributing to morbidity. Surgical repair using mesh placement has become the standard of care, with ongoing debates regarding the optimal anatomical plane for mesh placement. Even though the retrorectus plane advocated by Rives and Stoppa has become the choice of plane for most surgeons, it is not without recurrence. This prospective observational study compares the outcomes of incisional hernia repair using anterectus versus retrorectus mesh placement techniques.

Patients and methods

A total of 60 patients were enrolled from April 1, 2022, to April 1, 2024, at NRI Medical College & General Hospital, Vijayawada, India, divided equally into two groups. Group A included patients who underwent retrorectus hernioplasty, while Group B had anterectus hernioplasty. Parameters evaluated include epidemiological data, defect size, content, mesh size, operative time, postoperative pain, drain output, postoperative complications, hospital stay, recovery time, and recurrence rates.

Results

Results demonstrated a statistically significant advantage of the retrorectus approach with reduced operative time (160 ± 16 min vs. 216 ± 28 min; p < 0.0001), lower postoperative pain scores, decreased drain output, shorter hospital stays (5.6 ± 0.6 days vs. 15.7 ± 6.6 days; p < 0.0001), and fewer wound complications after a mean follow-up period of (17.4 +/- 4.7) months in group A and (18.3+/- 4.7) months in group B. Neither group had recurrences during the follow-up period.

Conclusions

This study concludes that retrorectus hernioplasty is superior to anterectus hernioplasty with less postoperative morbidity, shorter hospital stays, and accelerated patient recovery.

## Introduction

Incisional hernias arise through a defect in the musculofascial layers of the abdominal wall at a previous incision site. They occur in 10-50% of laparotomy incisions and 1-5% of laparoscopic port-site incisions. With an incidence of 7.4-11%, incisional hernia is a common surgical problem contributing to major morbidity and mortality [1,2].

All incisional hernias occurring between the lateral margins of both rectus sheaths are referred to as midline incisional hernias [3], and hernias occurring lateral to the lateral margin of rectus sheaths are known as lateral incisional hernias. These can be classified as M1 to M5 according to the European Hernia Society, and the patients in this study are M1 and M4. Suture repair of incisional hernias has a high recurrence rate of 63% [4]. Therefore, it is not recommended for routine practice, and currently, surgical treatment with prosthetic mesh is the standard method of repair [5]. As we understand the pathogenesis of hernia, we can appreciate the evolution of repair methods in both open and laparoscopic methods.

A recent classification by Parker et al. [6] describes the different planes in the abdominal wall for mesh placement. In the open surgical approach, mesh placement can be performed in various anatomical planes: intraperitoneally or extraperitoneally behind the rectus muscle in the retrorectus plane [7], anterior to the rectus muscle in the anterectus plane [8], or in the subcutaneous plane as in the on-lay technique [9].

It is understood that the deeper the plane of mesh placement, the lower the recurrence [10]. Superficially placed mesh in on-lay repair has a high chance of mesh exposure and infection secondary to wound infection, leading to a high recurrence rate [11]. Intraperitoneally placed mesh in direct contact with the intra-abdominal organs is prone to complications such as bowel obstruction secondary to adhesions, erosion into adjacent organs, and bowel fistulation [12].

The retrorectus plane is the current plane of choice for many surgeons in both open and laparoscopic approaches [13]. This study aims to prospectively determine whether anterectus mesh placement offers superior clinical outcomes compared to retrorectus mesh placement in open midline incisional hernia repairs in adults, with a focus on operative time, postoperative morbidity, hospital stay, and recurrence.

## Materials and methods

Study design and setting

This was a prospective observational study conducted in the Department of General Surgery at NRI Medical College & General Hospital, Vijayawada, India, in order to compare retrorectus versus anterectus fixation method for incisional hernia repair. The study was carried out over a period of two years from April 1, 2022, to April 1, 2024, and included patients undergoing elective incisional hernia repair. This study was approved by the Institutional Ethics Committee of NRI Medical College & General Hospital under protocol no. IEC PG60/Gen Surg4/2021-22.

Study sample

A total of 60 patients were included in the study, with equal allocation of 30 patients to each group. Group A comprised patients who underwent retrorectus hernioplasty, while Group B included those who underwent anterectus hernioplasty. The inclusion and exclusion criteria are summarized in Table 1. Patients meeting the eligibility criteria were assigned to consultant surgeons based on their outpatient department (OPD) visit, following a method of simple random sampling with blinding applied during the selection process. Surgeon A routinely performed retrorectus hernioplasty, whereas Surgeon B performed anterectus hernioplasty.

**Table 1:** Inclusion and exclusion criteria of the study

Inclusion	Exclusion
Patients aged 18-60	Patients with lateral incisional hernias
Patients undergoing elective incisional hernia repair via anterectus or retrorectus technique	Patients undergoing laparoscopic hernia repair
	Patients with immunocompromised states (diabetes, HIV/AIDS, malignancy, TB)

Methodology

**Figure 1:**
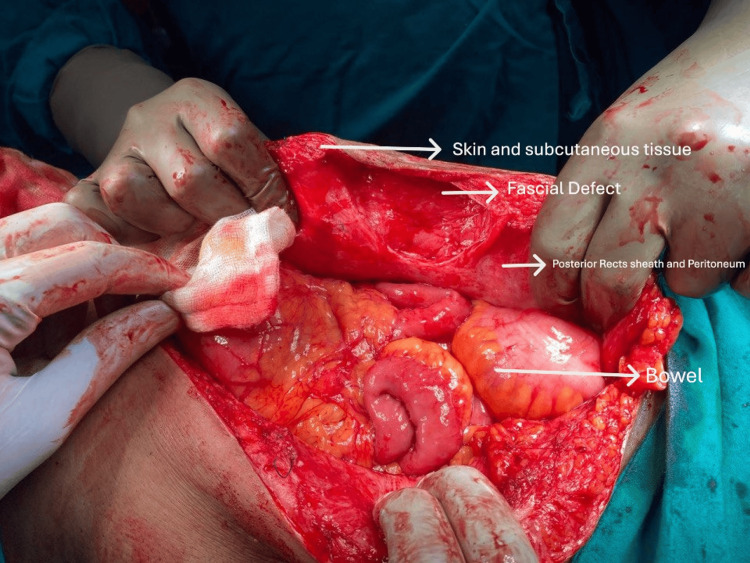
Hernia defect

**Figure 2:**
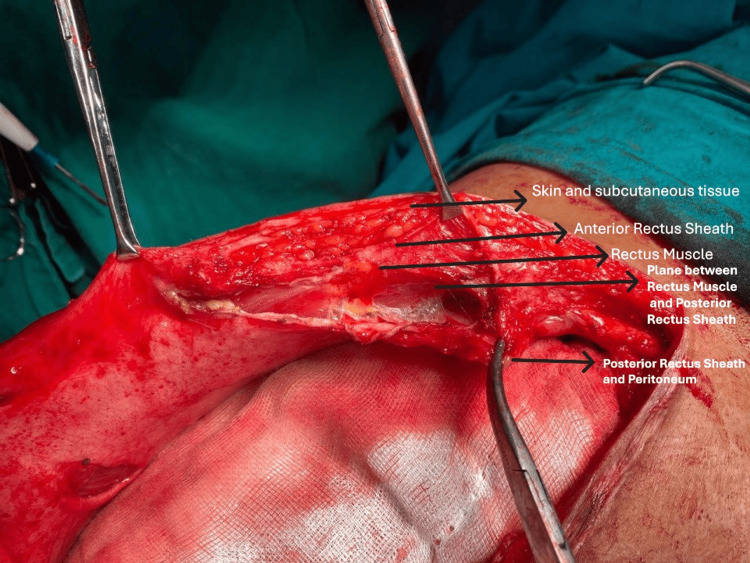
Retrorectus plane creation between the rectus muscle and posterior rectus sheath

**Figure 3:**
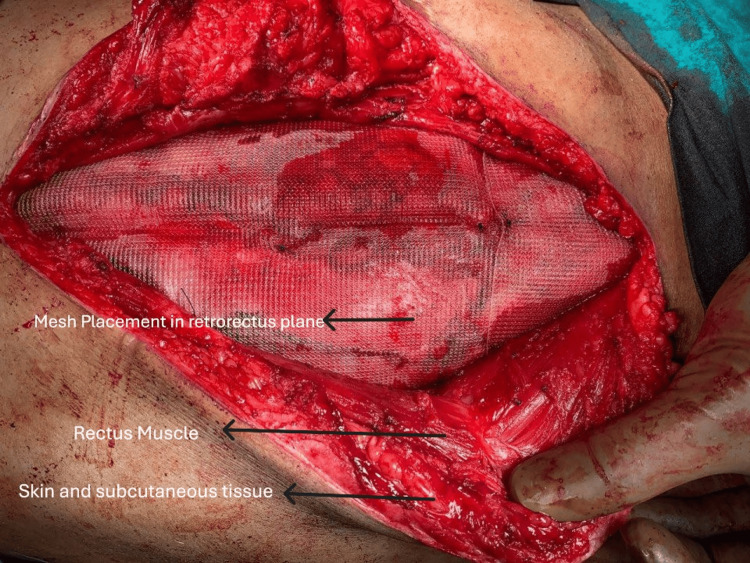
Mesh placement in the retrorectus plane

**Figure 4:**
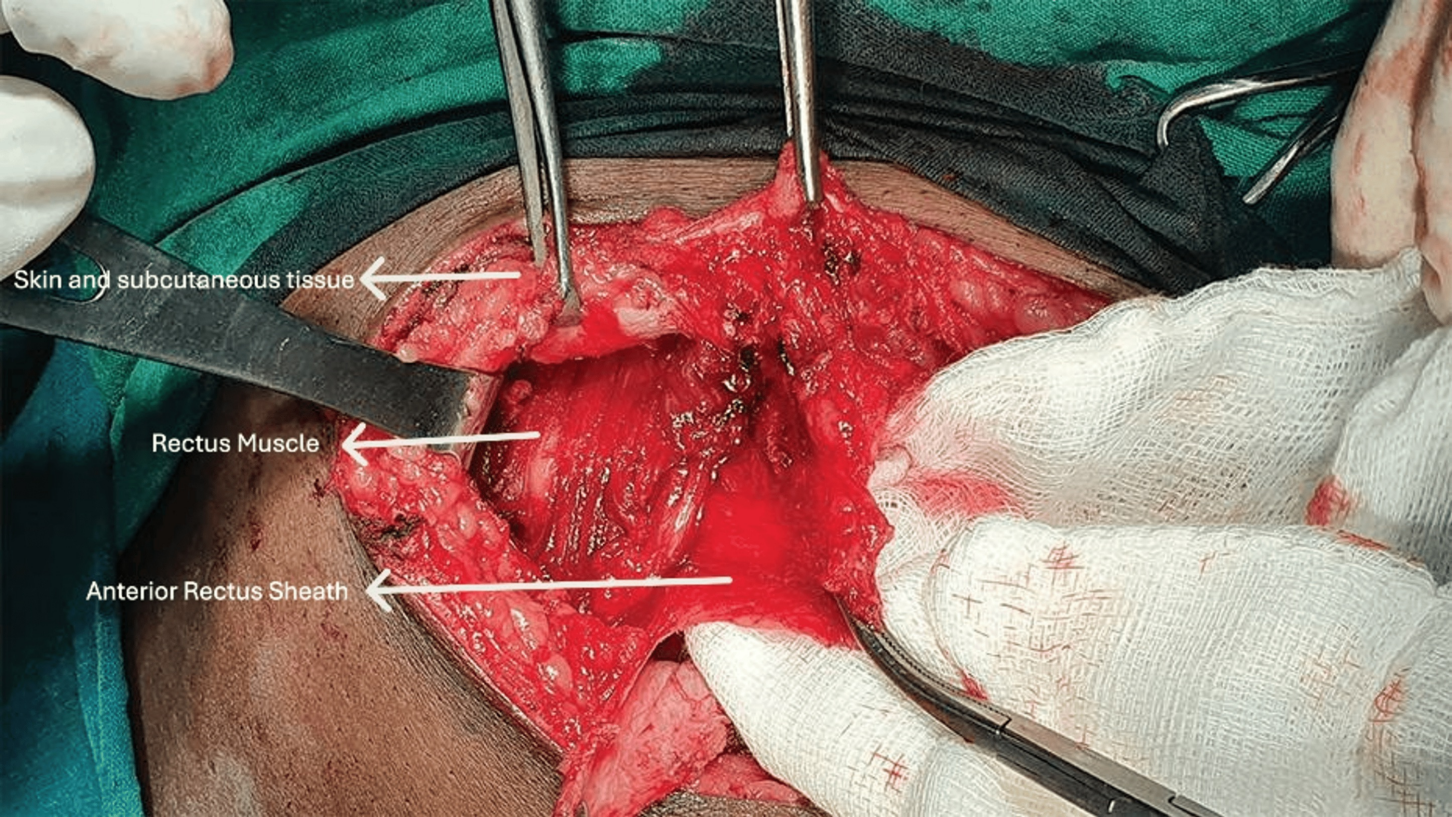
Anterectus plane creation between the rectus muscle and anterior rectus sheath

**Figure 5:**
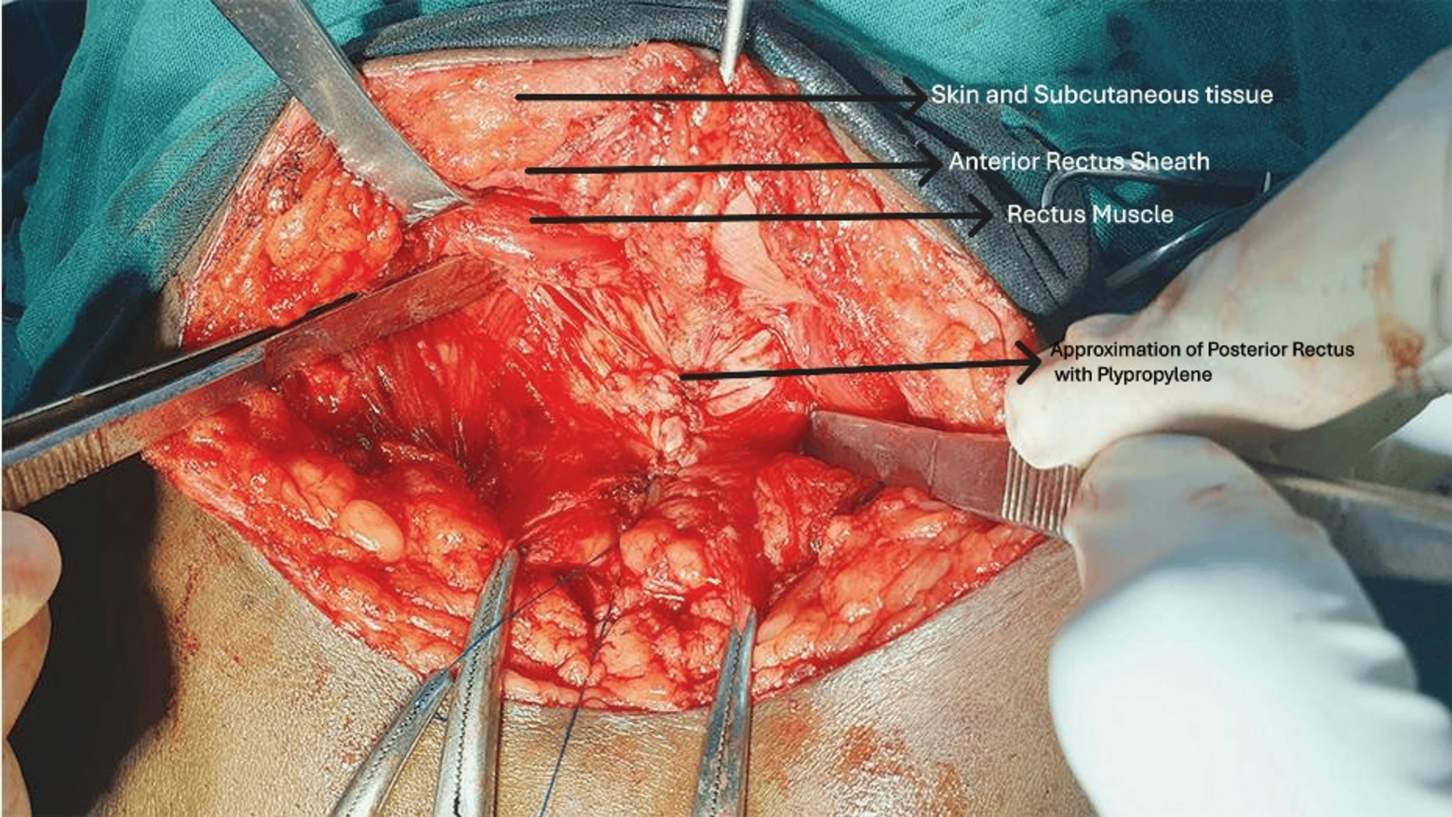
Closure of the posterior rectus sheath

**Figure 6:**
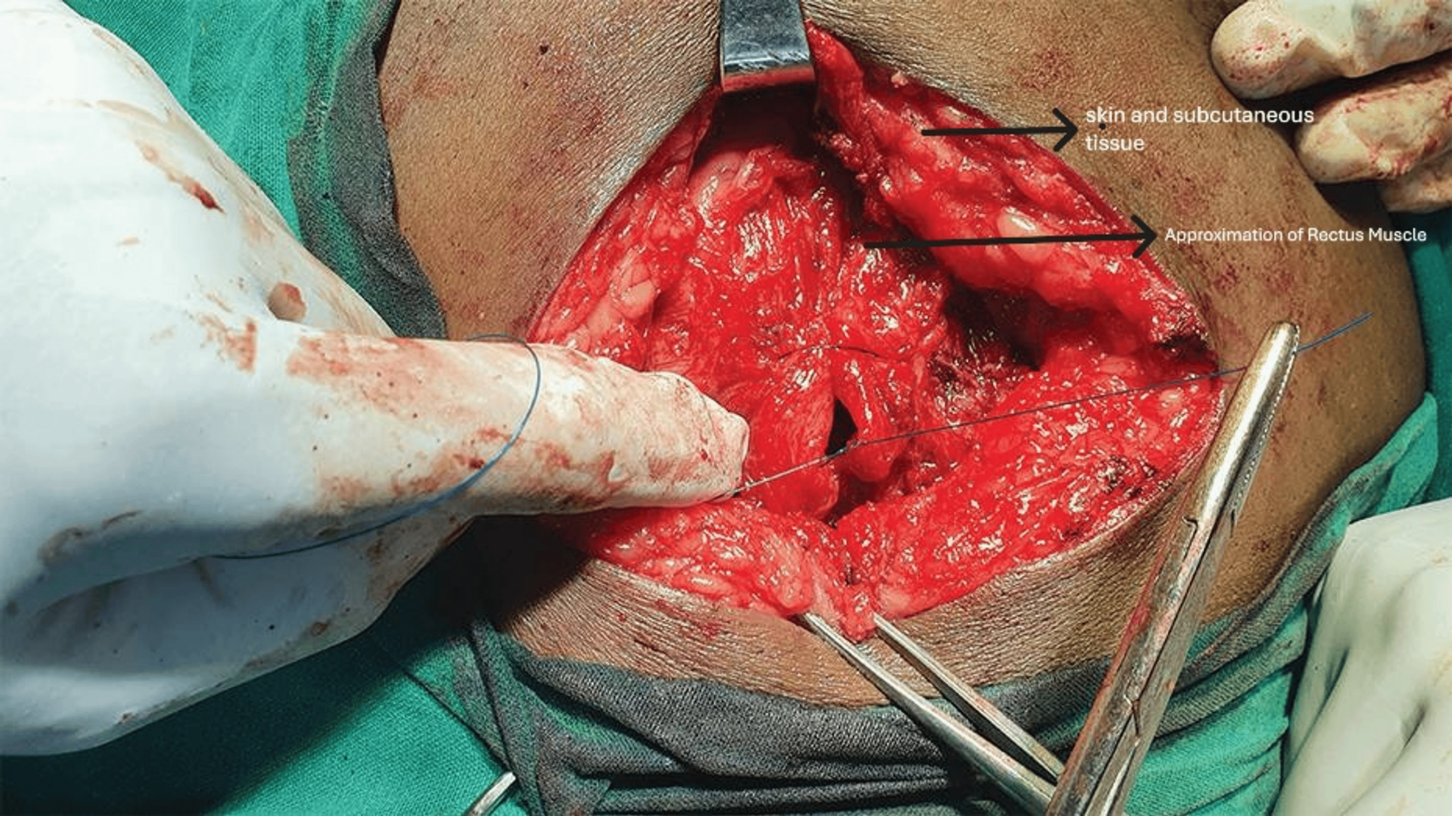
Approximation of the rectus muscle

**Figure 7:**
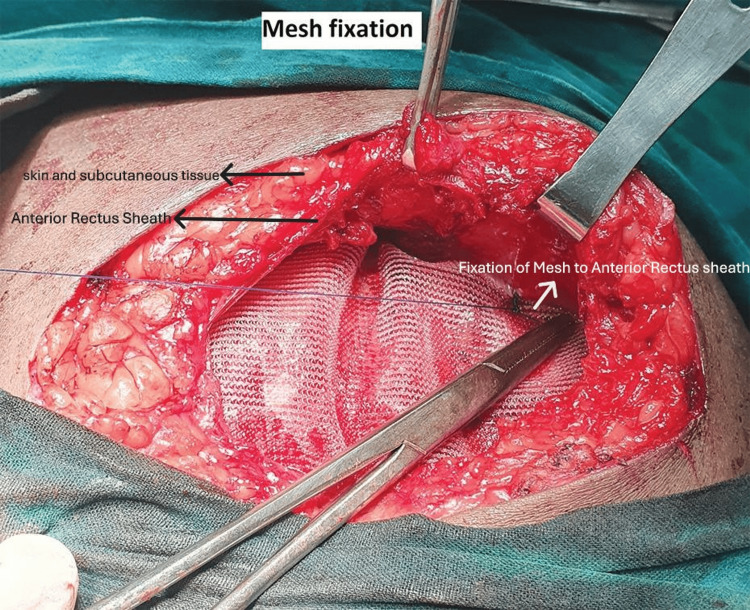
Mesh fixation

All surgeries were performed under either general or spinal anaesthesia by consultant surgeons using standard aseptic precautions. Patients undergoing hernioplasty via the retrorectus or anterectus approach were enrolled in a 1:1 ratio and assigned to Group A (retrorectus) and Group B (anterectus). Both primary and recurrent incisional hernias are classified as R0, R1, R2. Epidemiological, clinical, and radiological data were collected for all participants. Intraoperative findings were also documented, including the number of defects, size and area of each defect, hernia contents, mesh size, whether mesh fixation was performed, and the duration of surgery. The stepwise technique of retrorectus mesh fixation is illustrated in Figures 1-3 [14], and the surgical steps for anterectus mesh fixation are shown in Figures 4-7 [14].

A lightweight, macroporous, synthetic, non-absorbable polypropylene mesh was used for all patients in both groups. Mesh fixation was performed using an eight-point technique, anchoring the mesh at the 3, 6, 9, and 12 o’clock positions, as well as at all four corners to ensure secure placement.

Statistical analysis was conducted using Student’s t-test for continuous variables and the Chi-square test for categorical variables. All calculations were performed using the online tool socscistatistics.com. A p-value of less than 0.05 was considered statistically significant.

Postoperative pain was evaluated using a visual analogue scale (VAS) on postoperative days 3, 5, 8, 14, and 30. Wound inspections were also performed on the same days to assess for complications such as seroma, hematoma, wound dehiscence, surgical site infection (SSI), mesh infection, and skin flap necrosis. SSIs were identified based on clinical signs, including fever, tenderness, erythema, discharge, and abnormal drain output.

Drain was inserted for all patients, and output was measured every day until the drainage decreased to <15 ml/day over three consecutive days or stopped. Comparison was made out of the study variables observed in both the anterectus and retrorectus hernioplasty groups and further assessed. The variables evaluated in both groups are listed in Table 2.

**Table 2:** Study variables

Demographics	Hernia characteristics	Mesh	Postoperative outcomes				
			Drain	Wound complications	Patient-related outcomes	Length of hospital stay (days)	Recurrence of hernia
Age	Number of defects	Size (cm²)	a. Output (ml)	a. Seroma	a. Pain score (VAS)		
Gender	Surface area of defect (cm²)	Fixation	b. No. of days in situ	b. Hematoma	b. Time to return to daily activities		
				c. Wound dehiscence			
				d. Skin flap necrosis			
				e. Surgical site infection			
				f. Mesh infection			

The primary outcome of the study is hernia recurrence at two-year follow-up, while the secondary outcomes are operative time, postoperative complications (seroma, SSI), length of hospital stay, and postoperative pain. 

## Results

This was a prospective observational study conducted in the Department of General Surgery at NRI Medical College & General Hospital, Vijayawada, over two years. A detailed comparison of patient characteristics, operative details, and outcomes between the two groups is presented in Tables 3-13.

**Table 3:** Age and gender distribution

	Group A		Group B	
	Number	%	Number	%
Age group (in years)				
21-30	3	10%	5	16.6%
31-40	7	23.3%	10	33.3%
41-50	9	30%	7	23.3%
51-60	6	20%	4	13.3%
61-70	3	10%	4	13.3%
71-80	2	6.6%	0	0%
Mean age	47		44	
Gender				
Female	27	90%	28	93.3%
Male	3	10%	2	6.3%

**Table 4:** Distribution of patients according to previous surgeries CABG: coronary artery bypass grafting, LSCS: lower-segment caesarean section, TAH: total abdominal hysterectomy

Previous surgeries	Group A	Group B	Total	%
Tubectomy	10 (33.3%)	8 (26.6%)	18	30%
LSCS with midline incision	8 (26.6%)	12 (40%)	20	33.3%
TAH with midline incision	6 (20%)	3 (10%)	9	15%
Incisional hernia repair	2	3	5	8.3%
CABG	-	1	1	1.6%
Laparotomy	4	2	6	10%

**Table 5:** Distribution of patients according to primary and recurrent hernia

		Group A	Group B
Primary (R0)		28	27
Recurrent	(R1)	1	3
	(R2)	1	0
Total		30	30

**Table 6:** Distribution of patients according to the number of defects and defect area (cm²)

		Group A	Group B	P value
1 defect		24 (80%)	14 (46%)	
>1 defects		6 (20%)	16 (54%)	
Defect area	<15 cm^2^	16	10	
	15-30 cm^2^	9	8	
	>30 cm^2^	5	12	
Mean defect area		20 ± 23 cm^2^	36 ± 42 cm^2^	0.07

**Table 7:** Distribution of patients according to mesh size (cm2)

Mesh size	Group A	Group B	p-value
≤150 cm^2^	7	3	
150 to ≤ 225 cm^2^	18	20	
≥225 cm^2^	4	7	
Mean mesh size	248 ± 83.5 cm^2^	264.3 ± 151 cm^2^	0.6

**Table 8:** Distribution of patients according to the duration of surgery (min)

Duration of surgery (min)	Group A	Group B	P-value
≤150 (min)	19	1	
150-180 (min)	11	7	
180-210 (min)	0	8	
210-240 (min)	0	14	
Mean	160 ± 16 min (2 hr 30 min)	216 ± 28 min (3 hr 16 min)	<0.0001

**Table 9:** Distribution of patients according to postoperative pain

Mean postoperative pain score	Group A	Group B	P-value
Day 3	6.7 ± 0.7	7.2 ± 0.5	0.002
Day 5	4.9 ± 0.6	5.5 ± 1.19	0.016
Day 8	3.7 ± 0.79	4.1 ± 1.02	0.09
Day 14	2.9	2.9	
Day 30	1.4 ± 0.4	1.3 ± 0.3	

**Table 10:** Distribution of patients according to post-op drain output and average no. of days with drain in situ

	Group A	Group B	
Days	Mean drain output (ml)		P-value
Day 1	62.3 ± 19.7	96 ± 18.6	<0.0001
Day 2	36 ± 13.5	55 ± 14	<0.0001
Day 3	23.8 ± 16.9	33 ± 12	0.018
Day 4	14.8 ± 10	11.9 ± 8.8	0.2
Day 5	10 ± 4.8	18 ± 9.6	0.0001
Day 6	9.6	9.6	
Day 7	8.3 ± 2.8	8.5 ± 2.3	0.7
Mean no. of days with drain in situ			
Mean no. of days in situ (Group A) 5.5 ± 0.6		Mean no. of days in situ (Group B) 7 ± 0	

**Table 11:** Distribution of patients according to the mean duration of postoperative hospital stay

Days in hospital	Group A	Group B	P-value
5	17		
6	7		
7	3		
8			
10			
12		17	
14		3	
15		5	
30		5	
Mean	5.6 ± 0.6	15.7 ± 6.6	<0.0001

**Table 12:** Distribution of patients according to postoperative complications

Complications	Group A		Group B		P-value
	Number	%	Number	%	
No	28	93.3%	25	83.3%	0.22
Yes	2	6.6%	5	16.6%	
Type of complication					
Seroma	1	3.3%	5	16.6%	0.08
Hematoma	-	-	-	-	
Wound dehiscence (WD)	2	6.6%	5	16.6%	0.23
Skin flap necrosis (SFN)	1	3.3%	5	16.6%	0.08
SSI	1	3.3%	-	0	
Mesh infection (MI)	-	-	-	-	

**Table 13:** Distribution of patients according to their followup period

Duration	No. of patients	
	Group A	Group B
10-12 months	5	4
12-18 months	13	9
18-25 months	12	17
Mean followup	17.4 ± 4.7	18.3 ± 4.7

The majority of patients presented in the third and fourth decades of life, with the mean age of 47 years in Group A (retrorectus) and 44 years in Group B (anterectus). Female predominance was noted in both groups, i.e., 27 (90%) in Group A (M:F = 1:9) and 28 (93.3%) in Group B (M:F = 1:12). The distribution of patients by age and gender is detailed in Table 3.

The most common preceding surgeries were tubectomy, lower-segment caesarean section (LSCS), and total abdominal hysterectomy (TAH), accounting for 10 (33.3%), eight (26.6%), and six (20%) of cases in Group A and eight (26.6%), 12 (40%), and three (10%) in Group B, respectively. These distributions are detailed in Table 4.

Primary and recurrent incisional hernias are labelled as R0, R1, R2, etc. according to the number of previous repairs. Of the total 60 patients, five had recurrent hernias: two in Group A and three in Group B. Out of the total five patients in the study, four patients had recurrent hernias (R1) and one with a re-recurrent hernia (R2). Among the four patients of [R1], three had undergone anterectus hernioplasty and one patient had undergone retrorectus hernioplasty. One patient of re-recurrent incisional hernia [R2] had undergone retrorectus hernioplasty. This breakdown is presented in Table 5. 

In terms of the number of defects and defect area (cm²), in Group A, 24 (80%) patients had a single defect, while six (20%) had multiple defects. In Group B, 14 (46%) had a single defect and 16 (54%) had multiple defects. The defect area is calculated for both single and multiple defects and assessed. There is no difference in mean defect area in both groups, with a p-value of 0.07. Refer to Table 6 for full details.

The size of the meshes used in both groups is calculated. The mean mesh size was 248 ± 83.5 cm² in Group A and 264.3 ± 151 cm² in Group B (p = 0.6), with no statistically significant difference in the mean mesh size in both groups, with a p-value of 0.6. The mesh size (cm²) distribution across groups is shown in Table 7.

The mean operative time was significantly longer in Group B (216 ± 28 minutes) compared to Group A (160 ± 16 minutes; p < 0.0001), likely due to the more technically demanding nature of the anterectus approach. The detailed distribution of operative durations (min) is provided in Table 8.

Group B patients reported significantly higher pain scores on postoperative days 3 and 5 (VAS: 7.2 vs. 6.7 on day 3, p = 0.002; 5.5 vs. 4.9 on day 5, p = 0.016). Pain scores on other days were comparable. Pain trends are summarized in Table 9.

Drain was placed for all the patients in both groups, and the output (ml) was recorded every day. Drain output was significantly higher in Group B on days 1-3 (p < 0.0001, < 0.0001, and 0.018, respectively). The patients of Group A had an early drain removal, and for all the patients of Group B, the drain was removed on postoperative day 7. Hence, the patients of Group B had an increased average number of days with a drain in situ. These findings are shown in Table 10.

The majority of patients in Group B were discharged after suture removal. Hence, the patients of Group B had significantly longer mean postoperative hospital stay with a significant p-value of <0.0001. The detailed duration of stay data is provided in Table 11.

In Group A, 28 (93.3%) patients had an uneventful postoperative course, whereas the other two (6.6%) patients had wound dehiscence secondary to surgical site infection and skin flap necrosis, which were also managed conservatively. Twenty-five (83.3%) patients of Group B had an uneventful postoperative course, while the other five (16.6%) patients had seroma leading to skin flap necrosis and wound dehiscence, which were managed conservatively with debridement and regular dressings. However, statistically, there is no difference in the incidence of postoperative complications in both groups, with p-values of 0.08, 0.23, and 0.08 for seroma, wound dehiscence, and SSI, respectively. Table 12 outlines the postoperative complication rates and types.

All the patients were followed regularly at one-month intervals, and none of the patients in both groups had any incidence of recurrence during the follow-up period. The mean follow-up duration was 17.4 ± 4.7 months for Group A and 18.3 ± 4.7 months for Group B, as presented in Table 13.

## Discussion

This prospective observational study from April 2022 to 2024 aimed to compare the clinical outcomes of anterectus and retrorectus mesh placement techniques in the surgical repair of midline incisional hernias. Cases that fulfilled the inclusion and exclusion criteria were followed up postoperatively; this included 60 patients who were evaluated over a two-year period, with 30 patients allocated to each surgical technique.

In our study, the patients of incisional hernia commonly presented in their third and fourth decades of life, while the mean age of presentation was slightly lower in the anterectus group (44 years) compared to the retrorectus group (47 years). These findings are consistent with previously published RCTs by Venclauskas et al. [15], Demetrashvili et al. [16], and Sevinc et al. [17], who reported higher mean ages of 53, 59.6, and 55.9 years, respectively, for retrorectus hernioplasty and consistent for anterectus hernioplasty with Bandlamudi [14] and Miyake et al. [18], who reported mean ages of 46 and 73 years, respectively. A pronounced female predominance was observed in both groups secondary to their previous gynecological procedures. The prevalence in females also aligns with the existing literature. 

Statistically, in terms of intraoperative parameters, there was no significant difference found in the mean defect area or mesh size between the two groups. However, the operative time was significantly longer in the anterectus group (mean: 221 ± 39 minutes) compared to the retrorectus group (160 ± 16 minutes, p < 0.0001), likely due to the technical complexity of dissecting the anterectus plane. Postoperative recovery favored the retrorectus approach as the patients in the anterectus group experienced higher pain scores on postoperative days 3 and 5, with statistically significant differences (p = 0.002 and p = 0.016, respectively). As stated by Miyake et al., the cranial and caudal ends of the mesh are sutured to the fascia, which may have contributed to the postoperative pain. Therefore, reducing the number of sutures may reduce the pain experienced by patients [18].

Similarly, postoperative drain output is significantly higher in the anterectus group during the first three postoperative days with p-values of <0.0001, <0.0001, and 0.018, respectively. Drain removal occurred earlier in the retrorectus group, with a mean duration of 5.5 ± 0.6 days versus seven days in the anterectus group.

The duration of hospital stay was significantly shorter in the retrorectus group (5.6 ± 0.6 days) compared to the anterectus group (15.7 ± 6.6 days, p < 0.0001). These results are in agreement with existing studies, such as those by Venclauskas et al. [15] Demetrashvili et al. [16], and Sevinc et al. [17], i.e., 5.5 ± 1.6, 5.2 ± 2.4, and 3.52 ± 2.6, respectively, which reported shorter hospital stays following retrorectus repair. 

Wound complications were observed in two (6.6%) patients in the retrorectus group and five (16.6%) in the anterectus group. Although these differences were not statistically significant, they reflect a trend consistent with prior research in comparison with the RCTs of Venclauskas et el. [15], Demetrashvili et al. [16], and Sevinc et al. [17]. The rate of wound complications was 24%, 22.1%, and 8%, respectively. Notably, none of the patients in either group developed mesh infections. 

Wound-related complications in the anterectus group included seroma formation, skin flap necrosis, and wound dehiscence, which were managed conservatively. It can be observed from the results that the same five patients had all three complications mentioned above.** **Statistics for this group** **are comparable to the complication rates of 13% and 6.3% reported by Bandlamudi [14] and Miyake et al. [18] and better than Hodgman et al. [19], who reported 30% wound complications. These complications may be secondary to the superficial positioning of the mesh, and despite some patients requiring debridement, healing by secondary intention was successful. 

Our seroma formation rate was high in the anterectus group at five (16.6%) compared to Licheri et al. and Chevrel [20,21], which ranges from 6.29 to 11%. The aforementioned studies both used fibrin glue spray to fix the mesh, while we used suture alone, which may be a contributing factor. Although this has contributed to high drain output, it is difficult to ascertain whether seroma occurrence was secondary to creating a tissue plane or due to surgical techniques. However, studies have shown a biological mesh to increase prevalence. Tuveri et al. used a porcine small intestinal submucosa (SIS) and reported a seroma rate of 72% [22]. It is also worth noting that despite these complications, there was no increased hernia recurrence in this group.

In our study, the mean follow-up period was 17 months for the retrorectus group and 18 months for the anterectus group. During this time, no cases of hernia recurrence were observed in either group. We acknowledge that most recurrences occur at a median time of 2.5 years, and our follow-up period may be inadequate to detect the later hernia recurrences [23]. However, it's worth noting that patients often do not present for regular follow-up to the same surgical team.

Previous studies support these findings. Miyake et al. [18] reported that placing the mesh under the anterior rectus sheath is a safe and effective technique, with no recurrences and a wound complication rate of only 6.3%. Similarly, studies by Venclauskas et al. [14] and Sevinc et al. [16] observed recurrence rates of 2% following retrorectus repair, with follow-up durations of 12 and 36 months, respectively. Bandlamudi [17] and Miyake et al. [18] also reported low recurrence rates of 1.6% and 0% in their respective studies on anterectus repairs.

Overall, this study supports the superiority of retrorectus mesh placement in terms of operative time, postoperative morbidity, pain, hospital stay, and recovery, although both techniques demonstrated favorable long-term outcomes with no recurrence. The retrorectus technique, being more established, offers consistent results and may be preferred, especially where surgeon's experience and anatomical conditions permit. The anterectus approach, while effective, may involve a longer learning curve and should be considered in selected cases. A comparison of retrorectus hernioplasty outcomes with previous studies is provided in Table 14 and Table 15 compares the results of anterectus hernioplasty with the existing literature.

**Table 14:** Comparison of retrorectus hernioplasty with the existing literature

Study variable	Our study	Venclauskas et al. [15]	Demetrashvili et al. [16]	Sevinc et al. [17]
Age in years	47	53	59.6	55.9
M:F ratio	1.9	0.8:1	0.9:1	1:2
Postoperative hospital stay (In days)	5.6 ± 0.6	5.5 ± 1.6	5.2 ± 2.4	3.52 ± 2.6
Wound complications	6.6%	24%	22.1%	8%
Follow-up (In months)	17	12	-	3
Recurrence	0%	2%	2.6%	2%

**Table 15:** Comparison of anterectus hernioplasty with the existing literature

Study variable	Our study	Bandlamudi [14]	Miyake et al. [18]
Age in years	46	44	73
M:F ratio	1.9	1:6	1:0.6
Wound complications	16.6%	13%	6.3%
Follow-up (In months)	18	36	18
Recurrence	0%	1.6%	0%

Limitations

This study is a small-center observational study, and it has small sample size and short duration of follow-up.

Future recommendations

Other factors that could have been analyzed further are the patient’s morbidity status, including smoking and BMI. Our study consisted of patients who had no smoking history and some with a BMI>30 kg/m^2^, which can contribute to wound complications and increase the chance of recurrence. Although it is unknown whether these have contributed to the wound complications in our study, we recommend future studies to document and explore if any correlation is identified [24]. Future studies could also compare the outcomes of anterectus versus retrorectus using a laparoscopic approach to assess for any improvement in length of hospital stay and wound complications.

Despite mesh repair being the preferred method of incisional hernia repair, Ishimoto et al. have documented an anterior component separation (ACS) technique in combination with an anterior rectus abdominis sheath turnover flap. This method is preferred in those who undergo radiotherapy, where there are concerns of mesh exposure or infection. Ishimoto et al. described a single case study of a 51-year-old female who developed an incisional hernia secondary to a laparotomy for ovarian tumours. The patient was operated on using the above technique and found to have no recurrent incisional hernia 2.5 years post follow-up. It would be interesting for future studies to compare the efficacy of anterectus mesh placement versus the ACS technique, especially in incisional hernias secondary to malignancy resection surgeries [25].

## Conclusions

This prospective observational study demonstrates that retrorectus mesh placement offers several advantages over the anterectus technique in the surgical management of midline incisional hernias. Anterectus hernioplasty is a relatively newer technique with a longer learning curve. Although both techniques showed comparable long-term outcomes with zero recurrence during the follow-up period, the retrorectus approach proved to be more efficient with overall reduced morbidity.

The retrorectus technique should be considered the preferred approach for open mesh repair of midline incisional hernias, particularly in cases where anatomical and technical factors are favorable. However, the anterectus technique remains a valid alternative, especially in situations where the posterior rectus sheath is deficient or compromised.
